# 2027 The role of lysyl oxidase in systemic sclerosis-associated lung fibrosis

**DOI:** 10.1017/cts.2018.138

**Published:** 2018-11-21

**Authors:** Xinh-Xinh Nguyen, Tetsuya Nishimoto, Takahisa Takihara, Logan Mlakar, Ellen Riemer, Jonathan Heywood, Amy Bradshaw, Carol Feghali-Bostwick

**Affiliations:** Medical University of South Carolina

## Abstract

OBJECTIVES/SPECIFIC AIMS: Systemic sclerosis (SSc) is a connective tissue disease of unknown etiology characterized by progressive fibrosis of the skin and multiple visceral organs. Effective therapies for SSc are needed. Lysyl oxidase (LOX) is a copper-dependent amide oxidase that plays a critical role in the crosslinking of the extracellular matrix (ECM). In this study, we investigated the role of LOX in the pathophysiology of SSc. METHODS/STUDY POPULATION: LOX expression and protein levels were measured in lung tissues and primary fibroblasts from patients with SSc and healthy controls. The effects of recombinant LOX (rLOX) were measured in vitro in primary fibroblasts, ex vivo in human lung tissues and in vivo in mice given bleomycin in combination with rLOX. LOX levels and activity were evaluated in lung fibroblasts treated with an endostatin-derived peptide that ameliorates fibrosis and in mice treated with bleomycin in combination with the peptide. Further, to differentiate the crosslinking activity of LOX from other potential effects, primary human fibroblasts were cultured with rLOX in the presence of the inhibitor, beta-aminopropionitrile. The expression levels of ECM (collagen and fibronectin), pro-fibrotic factors (IL-6 and TGF-beta), and transcription factor (c-Fos) were examined by real-time PCR, ELISA, immunoblotting, or hydroxyproline assay. RESULTS/ANTICIPATED RESULTS: LOX mRNA was increased in lung tissues and matching fibroblasts of SSc patients. rLOX-induced ECM production in vitro and ex vivo in lung fibroblasts and in human lung tissues maintained in organ culture, respectively. Additionally, TGF-beta and bleomycin induced ECM production, LOX mRNA expression and activity. Endostatin peptide abrogated these effects. In vivo, rLOX synergistically exacerbated pulmonary fibrosis in bleomycin-treated mice. The inhibition of LOX catalytic activity by beta-aminopropionitrile failed to abrogate LOX-induced ECM production. LOX increased the production of IL-6. IL-6 neutralization blocked the effects of LOX. Further, LOX induced c-Fos expression and its nuclear localization. DISCUSSION/SIGNIFICANCE OF IMPACT: LOX expression and activity were increased with fibrosis in vitro, ex vivo, and in vivo. LOX induced fibrosis via increasing ECM, IL-6 and c-Fos translocation to the nucleus. These effects were independent of the crosslinking activity of LOX and mediated by IL-6. Our findings suggest that inhibition of LOX may be a viable option for the treatment of lung fibrosis. Further, the use of human lung in organ culture establishes the relevance of our findings to human disease.

